# Supplementary material for: Causal interplay between lactose intolerance and gut microbiota: a combined bidirectional Mendelian randomization and in vivo validation study
Source: Front Nutr. 2026 Jun 1;13:1803337. doi: 10.3389/fnut.2026.1803337 (PMC13265576; doi:10.3389/fnut.2026.1803337)
Supplement: Supplementary file 1 [file Data_Sheet_1.zip › supplementary materials/Forward/leave one out cross validation/ebi-a-GCST90027599.finngen_R12_E4_LACTONAS.pdf]

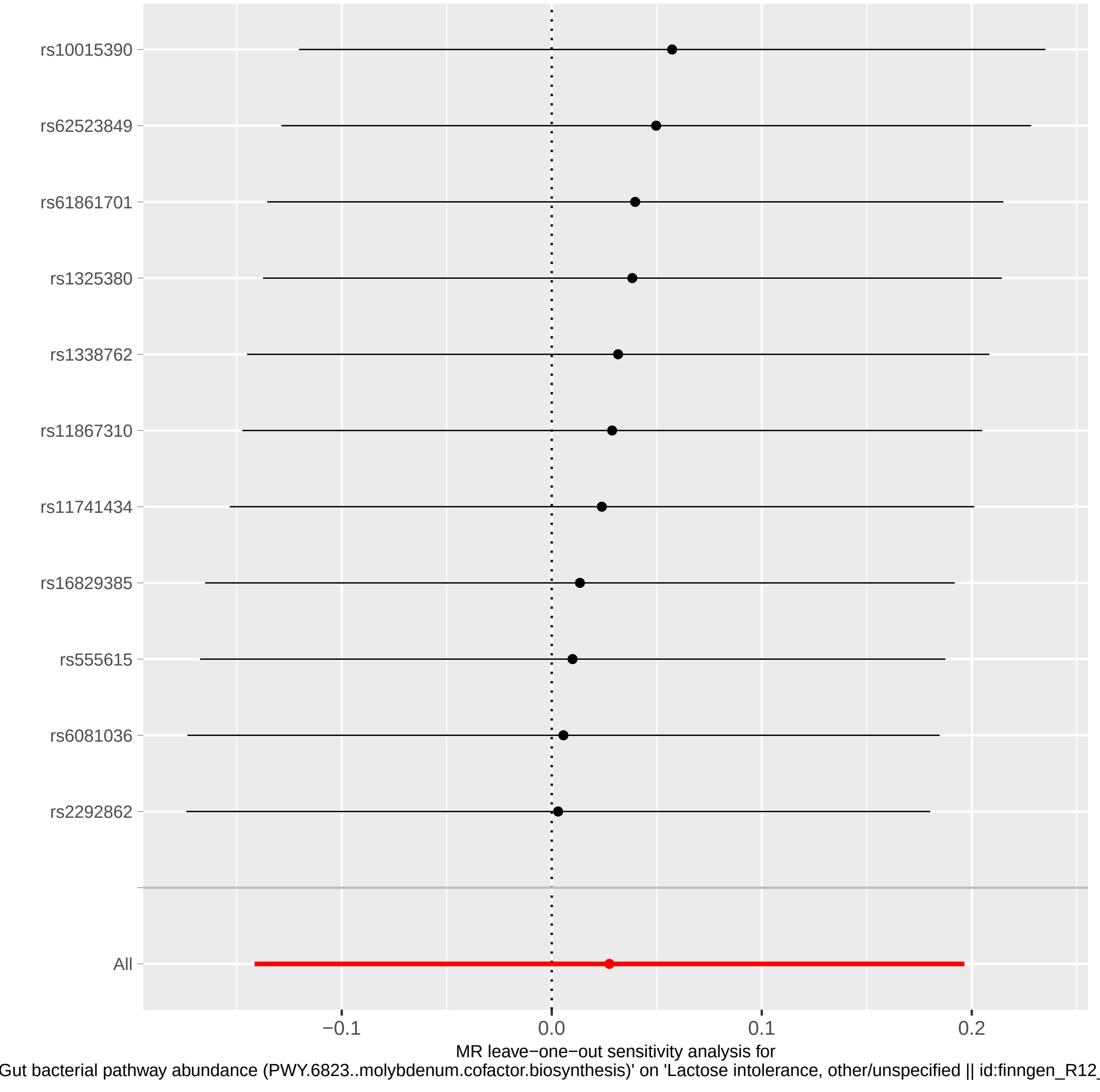

rs10015390
rs62523849
rs61861701
rs1325380
rs1338762
rs11867310
rs11741434
rs16829385
rs555615
rs6081036
rs2292862
All
−0.1
0.0
0.1
0.2
MR leave−one−out sensitivity analysis for
'Gut bacterial pathway abundance (PWY.6823..molybdenum.cofactor.biosynthesis)' on 'Lactose intolerance, other/unspecified || id:finngen_R12_
